# Cervical single open-door laminoplasty with or without local lateral mass screw fixation and fusion to treat cervical spinal cord injuries accompanied by segmental spinal canal stenosis

**DOI:** 10.3389/fsurg.2022.1050308

**Published:** 2023-01-04

**Authors:** Zihao Yu, Hongwei Xie, Ziyu Ouyang, Hua Zhang

**Affiliations:** ^1^ Department of Orthopedic Surgery, The Second Affiliated Hospital, Zhejiang University School of Medicine, Hangzhou, China; ^2^ Orthopedics Research Institute, Zhejiang University, Hangzhou, China; ^3^ Key Laboratory of Motor System Disease Research and Precision Therapy of Zhejiang Province, Hangzhou, China; ^4^ Clinical Research Center of Motor System Disease of Zhejiang Province, Hangzhou, China

**Keywords:** laminoplasty, lateral mass screw fixation, spinal cord injury, cervical spinal canal stenosis, neurological function

## Abstract

**Study Design:**

Retrospective.

**Objectives:**

To investigate the efficacy of cervical single open-door laminoplasty with and without local lateral mass screw fixation and fusion as treatments for cervical spinal cord injuries accompanied by multisegmental spinal canal stenosis.

**Setting:**

The Second Affiliated Hospital, School of Medicine, Zhejiang University.

**Methods:**

Of all enrolled patients, 42 formed a stable group who underwent cervical single open-door laminoplasty alone and 14 formed an unstable group who underwent the procedure combined with lateral mass screw fixation and fusion. Neurological function was evaluated before surgery, at discharge, and at final follow-up using the American Spinal Cord Injury Association (ASIA) impairment scale and the Japanese Orthopedic Association (JOA) score.

**Results:**

ASIA scores reflected improved neurological function in 52.5% of the stable group (15 with grade-D and 4 with grade-A injuries did not improve) and 45.5% of the unstable group (3 with grade-D and 3 with grade-A injuries did not improve). Postoperative JOA scores reflected 19.1% ± 21.6% improvement in the stable group and 18.6% ± 18.4% improvement in the unstable group (*P* > 0.05). Final follow-up JOA scores reflected 49.2% ± 31.7% improvement in the stable group and 47.1% ± 39.2% improvement in the unstable group (*P* > 0.05).

**Conclusions:**

Laminoplasty combined with local fusion aided the treatment of unstable cervical spinal cord injuries and spinal stenosis. Such stenosis is the main pathological factor causing multiple spinal cord compressions in patients with cervical spinal cord injuries.

## Introduction

Many high-energy injuries are osseous in nature; however, many low-energy spinal cord injuries in patients with pre-existing cervical stenosis are not associated with any fracture or other cause of instability. The treatment of central cord syndrome without associated instability remains controversial. Classically, nonsurgical treatment was considered to yield results similar to those of surgery (1). This position has recently been questioned; given the lack of instability, some authors have advocated non-fusion surgeries such as posterior laminoplasty (2–4). Fehlings et al. found that early surgical intervention improved neurological recovery in patients with cervical spinal cord injuries (5–7). However, the optimal surgical treatment, especially for patients with pre-existing stenosis and central cord syndrome, remains unclear. This study was a retrospective analysis of data from patients with cervical spinal cord injuries accompanied by multisegmental spinal canal stenosis treated *via* cervical single open-door laminoplasty. Additional intervertebral disc injuries, anterior longitudinal ligament injuries, and vertebral body avulsion fractures, when present, were stabilized *via* local lateral mass screw fixation and fusion in addition to laminoplasty. The improvements in neurological symptoms afforded by these treatments, the effects of surgical timing, and the risk factors for local instability were also investigated.

## Materials and methods

### Subjects

Between December 2014 and August 2017, 56 patients underwent surgery to treat traumatic cervical spinal cord injuries accompanied by multisegmental cervical spinal canal stenosis. The stenosis and cord injury were examined by magnetic resonance imaging (MRI). All patients evidenced greatly increased T2 signal changes in the spinal cord and cervical spinal canal stenosis [cervical sagittal diameter < 13 mm on MR images (8)] at three or more levels. Some stenoses were developmental [cross-sectional distance from the posterior edge of the vertebral body to the spinous process root < 13 mm on computed tomography (CT) images (9–11)]; other stenoses were attributable to the ossification of the posterior longitudinal ligament (OPLL), multilevel intervertebral disc herniation, and ligamenta flava folds. All patients were followed for at least 6 months. The exclusion criteria were vertebral compression, burst or facet fracture, cervical subluxation or dislocation, history of cervical spine surgery, upper cervical vertebral injury, and cervical kyphotic deformity. Patients who died or were lost to follow-up were excluded. We measured improvements using the American Spinal Cord Injury Association (ASIA) impairment scale and the Japanese Orthopedic Association (JOA) score.

### Surgical procedure

**Figure 1:**
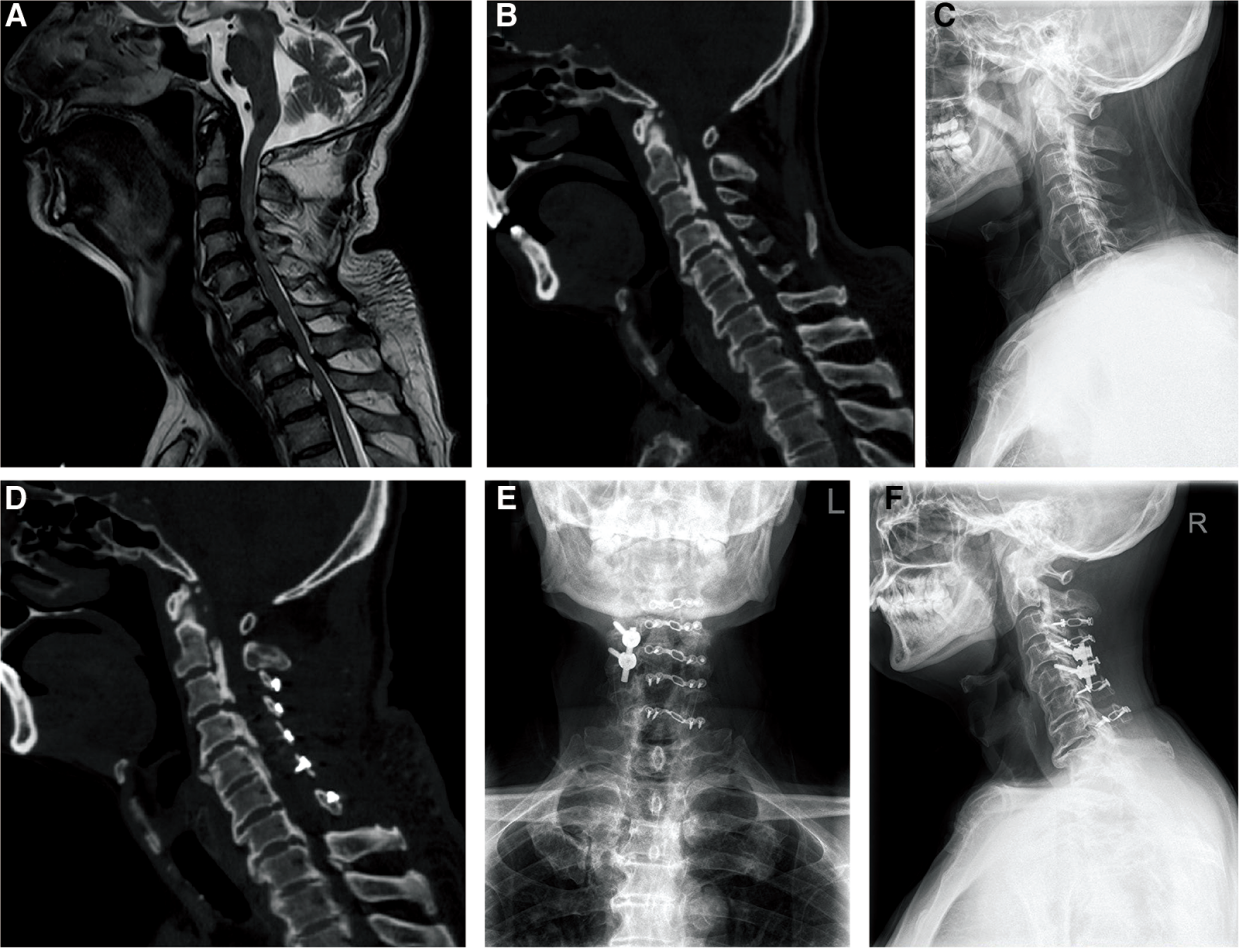
Imaging data of a typical case. A 66-year-old male patient with limb numbness caused by falling from height received single open-door laminoplasty and lateral mass screw fixation and fusion. (**A**) Preoperative MRI showed the rupture of C4/5 intervertebral disc and anterior longitudinal ligament, and the cervical spinal cord signal of C3–C6 increased on T2-weighted image. (**B**) Preoperative CT scan confirmed ossification of posterior longitudinal ligament and cervical spinal canal stenosis. (**C**) Preoperative radiograph. (**D**) Postoperative CT scan showed that the cervical spinal canal was enlarged and the effect of the operation was clear. (**E,F**). The anteroposterior and lateral radiographs 1 year after surgery suggested that the fixations were in good condition.

According to MRI and CT findings, the patients were divided into stable and unstable groups. Stability and instability were defined as the absence and presence, respectively, of intervertebral disc or anterior longitudinal ligament rupture or teardrop-like fracture at the anterior edge of the vertebral body. Simple cervical single open-door laminoplasty was indicated for the stable group. The open-door side was that of the most severe symptom. When symptoms on both sides were similar, we chose the side on which stenosis was more obvious on imaging. On the open-door side, fixation was performed using small titanium-alloy plates (ARCH Laminoplasty System, Synthes GmbH, Switzerland) to support the lamina and prevent postoperative “door closing.” Single open-door laminoplasty with local lateral mass screw fixation was indicated for the unstable group. Single open-door laminoplasty was used to treat the stenotic segment, and laminoplasty plus fixation with lateral mass screws was used to treat the local unstable segments. The lateral mass screws were placed on the hinge side and/or the open-door side. The facet joints of fusion segments were decorticated using a high-speed drill. Next, bone fragments from the autogenous spinous process were implanted in the facet joints. Methylprednisolone (80 mg QD) was prescribed for the first 2 postoperative days and was then reduced to 40 mg QD for the next 2 days to prevent spinal cord edema. Cefuroxime was prescribed at 2 g BID for 2 days postoperatively to prevent infection of the incision. The drainage tube was removed 48 h after surgery, or within 8 h when the drainage volume was <50 ml. After tube removal, the patients were encouraged to leave the bed and walk. The patients were told to protect their necks with a cervical collar for 2 weeks after surgery, and then to commence exercises that rehabilitated neck muscle function. Imaging data from a typical case are provided in Figure 1.

### Assessments

We recorded patient sex and age, cause of injury, time from injury to operation, duration of operation, amount of intraoperative blood loss, pathological type of cervical spinal stenosis, numbers and distributions of high-signal segments in the cervical spinal cord, and unstable segment pathological type and distribution. Patients' neurological status was evaluated before surgery, at discharge, and at the final follow-up using the ASIA impairment scale, and was classified as grades A–E based on symptom severity. Improvement was defined as at least one increment of improvement in the ASIA score at the final follow-up assessment. Neurological function was also evaluated using the JOA score before surgery, at discharge, and at the final follow-up. The JOA recovery rates at discharge and final follow-up were calculated using the Hirabayashi method as: [(postoperative JOA score—preoperative JOA score)/(17—preoperative JOA score)] × 100%.

### Statistical analysis

All statistical analyses were performed using SPSS software (ver. 12.0 for Windows; SPSS Inc., Chicago, IL, USA). We present means ± standard deviations. The unpaired independent-samples *t* test, Mann–Whitney *U* test, and chi-squared test were used, as appropriate, for group comparisons. *P* values < 0.05 were considered to be statistically significant.

## Results

The stable group comprised 42 patients (36 males and 6 females), including 1 patient who died and another who was lost to follow-up. The mean age was 59.1 ± 9.5 (range, 34–75) years, and the average time from injury to surgery was 10.1 ± 6.5 (range, 3–32) days. All patients in the stable group underwent cervical single open-door laminoplasty. Thirty-two patients required surgery from C3 to C7, eight patients required C3–C6 laminoplasty, and two patients required C4–C7 laminoplasty. The mean operation time was 128.6 ± 38.1 min and the average intraoperative blood loss was 136.3 ± 120.9 ml. The unstable group consisted of 14 patients (all male), including 3 who died during follow-up; thus, data from only 11 patients are provided in Table 1. The average age was 65.5 ± 10.2 (range, 49–84) years. The average time from injury to surgery was 7.7 ± 5.3 (range, 3–22) days. The operative details, including segments treated, are provided in Table 2. The average operative time was 134.5 ± 21.6 min and the average blood loss was 154.5 ± 90.7 ml. Patient age, the interval from injury to surgery, the operative time, and the amount of intraoperative blood loss did not differ between groups (all *P* > 0.05). Pre- and postoperative radiographic data from a typical case (from the unstable group) are provided in Figure 1.

**Table 1:** Basic information of patients in the unstable group.

Patient	Gender	Age (year)	Cause of injury	Unstable factors	Operations
1	M	71	Falling from standing	Rupture of the C4/5 disc and anterior longitudinal ligament.	C3–7 open-door laminoplasty combined with C4–5 bilateral lateral mass screws fixation and fusion.
2	M	84	Falling from standing	Rupture of the C5/6 disc.	C3–6 open-door laminoplasty combined with C4–5 unilateral (hinge side) lateral mass screws fixation and fusion.
3	M	72	Falling from a height	Rupture of the C3/4 disc.	C3–7 open-door laminoplasty combined with C3–4 unilateral (hinge side) lateral mass screws fixation and fusion.
4	M	63	Traffic	Rupture of the C3/4 disc and teardrop-like fracture at the anterior edge of C3 vertebral body.	C5–7 open-door laminoplasty, C3–4 laminectomy and bilateral lateral mass screws fixation and fusion.
5	M	66	Falling from a height	Rupture of the C4/5 disc and anterior longitudinal ligament.	C3–7 open-door laminoplasty combined with C4–5 unilateral (hinge side) lateral mass screws fixation and fusion.
6	M	49	Falling from a height	Rupture of the C3/4 disc and anterior longitudinal ligament.	C3–7 open-door laminoplasty combined with C3–4 unilateral (hinge side) lateral mass screws fixation and fusion.
7	M	68	Traffic	Rupture of the C3/4 disc and teardrop-like fracture at the anterior edge of C3 vertebral body.	C3–7 open-door laminoplasty combined with C3–4 unilateral (hinge side) lateral mass screws fixation and fusion.
8	M	54	Traffic	Rupture of the C6/7 disc and anterior longitudinal ligament.	C3–7 open-door laminoplasty combined with C6–7 unilateral (hinge side) lateral mass screws fixation and fusion.
9	M	53	Falling from standing	Rupture of the C4/5 and C5/6 discs.	C3–7 open-door laminoplasty combined with C4–6 unilateral (open side) lateral mass screws fixation and fusion.
10	M	72	Traffic	Rupture of the C4/5 disc and anterior longitudinal ligament.	C3–7 open-door laminoplasty combined with C4–5 unilateral (hinge side) lateral mass screws fixation and fusion.
11	M	69	Traffic	Rupture of the C4/5 disc and anterior longitudinal ligament.	C3–7 open-door laminoplasty combined with C4–5 unilateral (hinge side) lateral mass screws fixation and fusion.

**Table 2:** Comparison of demographic and surgical data between groups.

	Stable Group (*N* = 40)	Unstable Group (*N* = 11)	*P*-Value
Sex	Male 34 (85%) Female 6 (15%)	Male 11 (100%) Female 0 (0%)	0.171
Age (yearr)	59.1 ± 9.5	65.5 ± 10.2	0.078
Cause of injury	Traffic 16 (40%) Falling from a height 5 (12.5%) Falling from standing 19 (47.5%)	Traffic 5 (45.4%) Falling from a height 3 (27.3%) Falling from standing 3 (27.3%)	0.353
Injury-surgery interval (days)	10.1 ± 6.5	7.7 ± 5.3	0.229
Duration of operation (min)	128.6 ± 38.1	134.5 ± 21.6	0.510
Intraoperative blood loss (mL)	65.5 ± 10.2	154.5 ± 90.7	0.589

*P* < 0.05 was statistically significant.

High-energy injuries (falls from heights and car accidents) accounted for 52.5% of injuries in the stable group and 72.7% of injuries in the unstable group (*P* = 0.230). In the stable group, 64.3% of cervical canal stenoses were developmental; 9.5% of these cases were combined with OPLL. OPLL alone was observed in 21.4% of cases and simple multilevel disc herniation was observed in 14.3% of cases in this group. In the unstable group, all cervical spinal canal stenoses were developmental, and 35.7% of them were combined with OPLL (*P* = 0.03). Intervertebral disc injuries and vertebral body avulsion fractures were located principally in the regions of stress concentration in patients with multisegmental OPLL or intervertebral joint stiffness; the proportion of such patients in the unstable group was 57.1%.

Neurological function improvements are shown in Tables 3, 4. ASIA scores reflected improvement at the final follow-up assessment in 52.5% of patients in the stable group (15 patients with grade-D and 4 patients with grade-A function did not improve). Six of seven grade-D central spinal cord syndrome cases in this group remained grade D at the end of follow-up, accounting for 40.0% of unimproved cases. In the unstable group, 45.5% of patients improved (three patients each with grade-D and grade-A function did not). Postoperative JOA scores reflected 19.1% ± 21.6% improvement in the stable group and 18.6% ± 18.4% improvement in the unstable group (*P* > 0.05). JOA scores from the final follow-up assessments reflected 49.2% ± 31.7% improvement in the stable group and 47.1% ± 39.2% improvement in the unstable group. Improvements reflected by JOA scores obtained at discharge and final follow-up did not differ between groups. JOA scores improved in patients with grade-B–D neurological function according to the ASIA impairment scale.

**Table 3:** Comparison of the effect of surgical treatment between groups.

	Stable Group (*N* = 40)	Unstable Group (*N* = 11)	*P*-Value
ASIA scale before surgery	A 4 (10%) B 10 (25%) C 8 (20%) D 18 (45%) E 0 (0%)	A 3 (27.3%) B 1 (9.1%) C 4 (36.4%) D 3 (27.3%) E 0 (0%)	0.336
ASIA scale at discharge	A 4 (10%) B 9 (22.5%) C 7 (17.5%) D 20 (50%) E 0 (0%)	A 3 (27.3%) B 1 (9.1%) C 2 (18.2%) D 5 (45.4%) E 0 (0%)	0.580
ASIA scale at the final follow-up	A 4 (10%) B 0 (0%) C 6 (15%) D 27 (67.5%) E 3 (7.5%)	A 3 (27.3%) B 0 (0%) C 1 (9.1%) D 7 (63.6%) E 0 (0%)	0.244
Improvement at discharge	3/40 (7.5%)	2/11 (18.2%)	0.291
Improvement at the final follow-up	21/40 (52.5%)	5/11 (45.4%)	0.679
JOA score before surgery	5.2 ± 4.6	3.7 ± 4.1	0.324
JOA score at discharge	7.0 ± 5.9	5.9 ± 4.9	0.528
JOA score at the final follow-up	10.4 ± 5.4	9.5 ± 7.0	0.678
JOA recovery rate at discharge (%)	19.1 ± 21.6	18.6 ± 18.4	0.939
JOA recovery rate at the final follow-up (%)	49.2 ± 31.7	47.1 ± 39.2	0.869

*P* < 0.05 was statistically significant.

**Table 4:** Comparison of JOA score between pre-operation and post-operation.

	Preoperative JOA score	Postoperative JOA score	*P*-value
Stable group (*N* = 40)	5.2 ± 4.6	At discharge 7.0 ± 5.9 At the final follow-up 10.4 ± 5.4	<0.001 <0.001
Unstable group (*N* = 11)	3.7 ± 4.1	At discharge 5.9 ± 4.9 At the final follow-up 9.5 ± 7.0	0.018 0.005
Total cases (*N* = 51)	4.8 ± 4.0	At discharge 6.7 ± 4.6 At the final follow-up 10.2 ± 5.7	<0.001 <0.001

*P *< 0.05 was statistically significant.

## Discussion

We found that 73.2% of patients with cervical spinal cord injuries but no significant fracture or dislocation had developmental spinal canal stenosis (anteroposterior spinal canal diameter < 13 mm). The relationship between such stenosis and cervical spinal cord injury has been of concern to researchers. In asymptomatic populations, the incidences of cervical spinal cord compression and signal change are related closely to developmental spinal canal stenosis (9). Morishita et al. (11) showed that the kinematic properties of the cervical spine in patients with such stenosis (diameter < 13 mm) explain secondary pathological changes. This type of stenosis is a risk factor for cervical spondylosis (12, 13), and it is associated with a higher incidence of cervical spinal cord injury after mild trauma because it reduces the functional reserve space for the spinal cord. Aarabi et al. (14) reported a cervical spinal canal stenosis rate of 37.4% in a sample of 211 patients with central spinal cord injury syndrome. Aebli (15) found that Pavlov ratios < 0.7 and anteroposterior spinal canal diameter < 8 mm were risk factors for cervical spinal cord injury after mild trauma.

The use of surgery to treat cervical spinal cord injuries with no significant fracture or dislocation remains controversial. Similar outcomes of nonoperative and operative treatments have been reported (1, 16). However, other studies have shown that spinal canal decompression yields better results for patients with stable cervical spinal cord injuries and spinal canal stenosis (3, 4). In a retrospective study, Gu et al. (2) found more improvement, according to JOA and SF-36 scores, among patients with cervical spinal cord injuries and OPLL who underwent laminoplasty (*n* = 31) than among those who underwent nonoperative treatment (*n* = 29 cases). The degree of improvement in the JOA score at the final follow-up was about 50%. The JOA scores of all patients with grade-B–D function improved, whereas those of patients with grade-A function did not; no patient evidenced neurological deterioration after surgery. A prospective study on this topic would be difficult to conduct because the conditions and pathogenic factors of patients with spinal cord injuries are complex. Further research is needed.

The optimal timing of surgery remains controversial. Fehlings et al. (17) found no significant difference in neurological function improvement between early (≤24 h after injury) and late (>24 h after injury) surgery groups. Another study showed that operation within 8 h after injury was better than operation 8–24 h after injury (6). Early surgery is usually defined as that performed within 24 or 72 h. In China, however, primary hospitals lack experienced surgeons and the required surgical equipment, and patients are referred to regional medical centers. Most patients included in this study were referred from local primary hospitals, and some had other serious injuries, such as traumatic brain injuries and chest trauma. Thus, the performance of all emergency operations within 8 or 24 h is difficult. In a retrospective sample of 595 patients from 6 hospitals in China, 212 patients underwent early (<72 h after injury) surgery and 383 patients underwent late (≥72 h after injury) surgery (5). ICU stays were longer but hospital stays were shorter in the early surgery group than in the late surgery group, and the rates of complications (pneumonia, wound infection, and sepsis) did not differ between groups. More importantly, neurological improvements did not differ significantly between groups, but the neurological deterioration and mortality rates were higher in the early surgery group than in the late surgery group. The authors concluded that the performance of surgery >72 h after injury was safer. We also found that surgeries performed at this interval were effective; we noted no postoperative neurological deterioration in these cases. When resources are available and the patient's condition permits, emergency surgery (performed within 8 h of injury) may aid functional recovery in patients with severely impaired neurological function (ASIA grade A). Some authors have recommended emergency surgery for patients exhibiting locking of the bilateral cervical facets with incomplete paralysis or worsening neurological function (6).

A semi-hybrid surgical technique involving laminoplasty and internal screw fixation has been used to treat degenerative cervical diseases, including cervical spondylosis with kyphosis (18, 19) and cervical spondylosis (or OPLL) combined with the presence of unstable segments (20, 21). However, few reports on the use of this technique to treat traumatic cervical spinal cord injuries have been published (22–24). The technique not only reduces the need for operation *via* the anterior approach, but avoids the posterior scar formation and risk of C5 nerve root palsy associated with laminectomy and internal fixation (22, 25). One report describes the treatment of cervical spinal cord injuries in six patients in Korea *via* single open-door laminoplasty combined with internal fixation with unilateral lateral mass screws; the clinical results were good (22). As kyphosis was present, the authors used long-segment lateral mass screw fixation (i.e., with an average of five screws) to correct the cervical deformities. Some surgeons have combined laminoplasty with pedicle screw fixation to treat cervical spinal canal stenosis accompanied by unstable fractures (23, 24). We found that cervical single open-door laminoplasty combined with short-segment lateral mass screw fixation enabled adequate stabilization of local segment instabilities caused by intervertebral disc damage or avulsion fracture, and that the neurological improvement rate did not differ significantly between the stable and unstable groups. We found that many unstable segments were located in regions of stress concentration, such as areas of discontinuous ossification in patients with multisegmental OPLL or intervertebral joint stiffness. Thus, areas of stress concentration constitute a risk factor for local instability after mild trauma.

The limitations of this study include the small sample and slight variation in hybrid techniques employed, specifically the side and number of lateral mass screw implantations, according to surgeons’ preferences. More long-term or multicenter data are required. In addition, this study was retrospective and lacked a control group. The evidence does not yet reveal whether the type of operation or the injury-to-operation interval (> or <72 h) affects the neurological outcomes.

## Data Availability

The original contributions presented in the study are included in the article/Supplementary Material, further inquiries can be directed to the corresponding author/s.
